# Solution and Membrane Interaction Dynamics of *Mycobacterium tuberculosis* Fatty Acyl-CoA Synthetase FadD13

**DOI:** 10.1021/acs.biochem.0c00987

**Published:** 2021-04-29

**Authors:** Camilla A. K. Lundgren, Michael Lerche, Charlotta Norling, Martin Högbom

**Affiliations:** Department of Biochemistry and Biophysics, Stockholm University, 106 91 Stockholm, Sweden

## Abstract

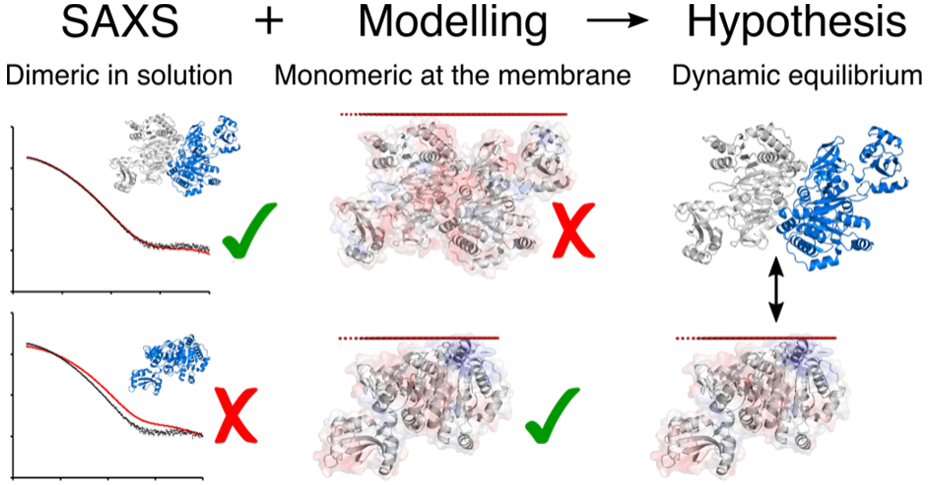

The very-long-chain fatty acyl-CoA synthetase FadD13 from *Mycobacterium tuberculosis* activates fatty acids for further use in mycobacterial lipid metabolism. FadD13 is a peripheral membrane protein, with both soluble and membrane-bound populations *in vivo*. The protein displays a distinct positively charged surface patch, suggested to be involved in membrane association. In this paper, we combine structural analysis with liposome co-flotation assays and membrane association modeling to gain a more comprehensive understanding of the mechanisms behind membrane association. We show that FadD13 has affinity for negatively charged lipids, such as cardiolipin. Addition of a fatty acid substrate to the liposomes increases the apparent affinity of FadD13, consistent with our previous hypothesis that FadD13 can utilize the membrane to harbor its very-long-chain fatty acyl substrates. In addition, we unambiguously show that FadD13 adopts a dimeric arrangement in solution. The dimer interface partly buries the positive surface patch, seemingly inconsistent with membrane binding. Notably, when cross-linking the dimer, it lost its ability to bind and co-migrate with liposomes. To better understand the dynamics of association, we utilized two mutant variants of FadD13, one in which the positively charged patch was altered to become more negative and one more hydrophobic. Both variants were predominantly monomeric in solution. The hydrophobic variant maintained the ability to bind to the membrane, whereas the negative variant did not. Taken together, our data indicate that FadD13 exists in a dynamic equilibrium between the dimer and monomer, where the monomeric state can adhere to the membrane via the positively charged surface patch.

*Mycobacterium tuberculosis* is a human pathogen and the causative agent of tuberculosis (TB). The spread of TB is considered a major global health crisis by the World Health Organization (WHO),^1^ and *M. tuberculosis* has been the leading cause of death by a single infectious agent since 2007. It is estimated that 1.7 billion people are infected with latent *M. tuberculosis* and at risk of developing active TB.^1^ Development of drug resistance of the pathogen is also a major cause of concern.^1^

Upon host entry, *M. tuberculosis* is engulfed by alveolar macrophages.^2,3^ Under normal circumstances, the phagosomes would acidify and fuse with lysosomes and their content would be targeted for destruction.^4^ However, *M. tuberculosis* has the ability to arrest acidification as well as fusion with lysosomes.^5−7^ The bacteria have evolved to maintain a state of chronicity and can survive in a quiescent state within the hostile environment of the host macrophages; such latent *M. tuberculosis* infection is particularly difficult to treat.^1,8^

*M. tuberculosis* is an unusual bacterium with a complex, highly impermeable, waxy cell envelope that confers a natural resistance to many antibiotics.^9,10^ This waxy cell envelope is key for *M. tuberculosis* survival and comprised of a complex layer of large mycolic acids.^9,10^ The biosynthesis machinery producing these complex lipids is an essential part of *M. tuberculosis* lipid metabolism.^11^ In addition, *M. tuberculosis* is mainly lipolytic *in vivo* and enzymes involved in lipid degradation are pivotal to survival.^7,12−15^

The lipid metabolism of *M. tuberculosis* is extensive, and *M. tuberculosis* encodes an astonishing 250 annotated genes devoted to lipid metabolism, 5 times the number of genes encoded by, e.g., *Escherichia coli* for the same purposes.^16^ However, the relationship and redundancy of the enzymes involved in lipid biosynthesis, uptake, and degradation are not well understood,^17^ and a more detailed understanding of these enzymes is vital for our understanding of this significant human pathogen.

Due to the metabolically inert nature of fatty acids, they have to be activated before they can enter into metabolic pathways.^18−21^ Thioesterification by coenzyme A (CoA), catalyzed by fatty acyl-CoA synthetases (FACS), is a common strategy for fatty acid activation.^18^ It is a two-step mechanism coupled to the cleavage of ATP to AMP and PP_i_. The final product is a fatty acyl chain thioesterified to a CoA moiety.

FACS belong to the large family of acyl activating enzymes (AAE)^21^ and are important node points in lipid metabolism because they activate fatty acids targeted to both degradative and biosynthetic pathways.^12,22,23^ They can be divided into five subfamilies based on the acyl-chain length of the preferred substrate: C_2_–C_4_ (short), C_4_–C_12_ (medium), C_12_–C_20_ (long), C_14_–C_24_ (“bubblegum”), and >C_20_ (very-long-chain).^23,24^ The long- and very-long-chain FACS are predominantly membrane-bound proteins.^24,25^

FACS are typically denoted FadD in bacteria, and *M. tuberculosis* encodes no fewer than 34 FadD enzymes, further emphasizing their central metabolic role.^8,26^ FadD13 is involved in fatty acid activation and shows a clear preference for very-long-chain fatty acids; the activity increases with acyl-chain length up to the limit tested (C_26_).^27^

The gene encoding FadD13 is part of the *mymA* operon (Rv3083–Rv3089), induced during acidic conditions similar to the environment within host macrophages.^28^ The *mymA* operon appears to be non-essential for growth in laboratory media but is required for growth inside host macrophages.^6^ It has been proposed that the *mymA*-encoded enzymes are important for mycomembrane assembly and remodeling of the membrane as a response to macrophage acidification.^29−31^

FadD13 shares the conserved AAE fold that is comprised of a large N-terminal domain and a smaller C-terminal domain proposed to move during catalysis.^32−34^ There is a large hydrophobic pocket extending from the active site, located at the interface between the domains, toward a distinct, positively charged patch on the surface of the N-terminal domain.^32^ Intriguingly, the hydrophobic pocket is not large enough to house the preferred very-long-chain fatty acyl substrates.

We have previously shown that FadD13 is a peripheral membrane protein with both soluble and membrane-bound populations *in vivo*. In addition, we hypothesized that the positive patch anchors the protein to the membrane, allowing the membrane to house part of the very-long-chain fatty acid substrate during catalysis.^32^ In this paper, we show that FadD13 has affinity for negatively charged lipids such as cardiolipin, and upon addition of a fatty acid substrate, the apparent affinity to the membrane is increased.

The literature is ambiguous with respect to the multimeric nature of FadD13 and other homologous FACS proteins, with reports of both monomeric and dimeric assemblies.^35−37^ Two possible dimeric arrangements can be inferred from the crystal lattice of the *M. tuberculosis* FadD13 crystal structure [Protein Data Bank (PDB) entry 3R44]. However, an analysis of the potential interaction surfaces gives no clear indication if either dimeric arrangement is stable in solution.

Here we show that FadD13 adopts a dimeric conformation in solution. Small-angle X-ray scattering (SAXS) defines the arrangement as the N-terminal dimer observed in the crystal lattice. However, this dimeric arrangement appears to be incompatible with membrane binding. Modeling the interaction using the Positioning of Proteins in Membrane (PPM) server^38^ confirms that only the FadD13 monomer can interact with a membrane. In addition, we show that cross-linking the FadD13 dimer abolishes membrane association and changing the properties of the positively charged surface patch has an effect on both dimerization and membrane association.

In summary, the data presented here strengthen the hypothesis that FadD13 exists in a dynamic equilibrium between a dimeric and monomeric form, where the monomer can adhere to the membrane via the positively charged surface patch. Once bound to the membrane, FadD13 can access and activate its very-long-chain fatty acyl substrates by utilizing the membrane to house the protruding acyl chains.

## Materials and Methods

### Cloning

The Rv3089 gene encoding the fatty acyl-CoA synthetase FadD13 (UniProt entry P9WQ37) was cloned from *M. tuberculosis* strain H37Rv into a pET28-TEV vector and fused with an N-terminal His_6_ tag. Mutant variants of wild-type FadD13 were created by a substitution of strategic residues at the positively charged surface patch. A hydrophobic variant (R9A/R17A/R195A/R197A/R244A) and a negative variant (R9E/R17D/R195E/Y196A/R197D/R244D) were created. The synthetic genes, provided by GeneArt, were moved into a pET-46 Ek/LIC vector and fused to an N-terminal His_6_ tag using ligation-free cloning.

### Expression

The plasmid vector with an insert (FadD13/hydrophobic variant/negative variant) was transformed into *E. coli* expression strains BL21(DE3) or T7 Express LysY. Terrific Broth supplemented with 50 μg/mL antibiotic (carbenicillin or tetracycline), 0.4% glycerol, and 0.01% Antifoam 204 was inoculated with a starter culture at a 1:100 dilution. The cultures were grown in a LEX bubbling system at 37 °C to a density OD_600_ of 2.0–2.5. Overexpression was induced by addition of 0.25 mM IPTG. The temperature was decreased to 20 °C, and the cultures were grown for an additional ∼18–20 h. The cells were harvested via centrifugation at 7500*g* for 15 min (JLA 8.1000, Beckman Coulter). The resulting cell pellet was transferred to 50 mL falcon tubes, frozen, and stored at −20 °C.

### Purification

Cells were thawed and resuspended in lysis buffer [50 mM Hepes, 5% glycerol, 600 mM NaCl, 1 mg/mL lysozyme, EDTA-free protease inhibitors (Roche), and 20 μg of DNase] and lysed in an Emulsiflex C3 system (Avestin Inc.) or by sonication (5 min cycles at 70% amplitude, 10 s on, 15 s off). The cell lysate was cleared by a 30 min centrifugation at 20000*g* (JA 14, Beckman Coulter). The supernatant was incubated with Ni^2+^-loaded NiNTA resin (Protino). The protein-loaded beads were washed with 30 CV of purification buffer [50 mM Hepes, 5% glycerol, and 600 mM NaCl] and 30 CV of purification buffer supplemented with 50 mM imidazole. The bound protein was eluted with 400 mM imidazole in purification buffer. The eluted protein was concentrated (VivaSpin 20, 30/50 kDa cutoff), and the concentrated sample was further purified by gel filtration chromatography (26/600 Superdex 200, 1 mL/min, or 16/60 Superdex 200, 0.5 mL/min, depending on sample volume). The fractions were pooled and concentrated (VivaSpin 20, 30/50 kDa cutoff) to approximately 19–25 mg/mg and flash-frozen in liquid nitrogen. If needed, the variants were run once more over a gel filtration column by collecting the monomer peak, concentrating it, and re-running it on the same column using the same conditions described above.

### Small-Angle X-ray Scattering Sample Preparation

The samples for SAXS were purified according to the description given above. The samples were thawed and re-run on a gel filtration column (16/60 Sephadex 200, 0.5 mL/min, or 10/300 Sephadex 200, 0.2 mL/min). Several samples from different parts of the peaks were analyzed.

### Small-Angle X-ray Scattering

SAXS measurements were carried out at 12.4 keV at Diamond Light Source beamline B21 in the momentum transfer (*q*) range of 0.0038–0.41 Å^–1^ [*q* = 4π sin(θ)/λ, where 2θ is the scattering angle] using a Pilatus 2M hybrid photon-counting detector (Dectris Ltd., Baden, Switzerland). A 30 μL sample of either FadD13, the negative or the hydrophobic variant, was loaded onto the sample capillary by the EMBL Arinax sample handling robot. Each data set comprised 18 exposures, for 180 s each. Identical buffer samples were measured before and after each protein measurement and used for background subtraction. Data merging, averaging, and subtracting were performed using the data processing tools in the EMBL-Hamburg ATSAS package.^39^ The radius of gyration (*R*_g_), maximum particle size (*D*_max_), and Porod volume were determined from tools in the PRIMUS program suite.^40^ Structures of dimer configurations were generated from symmetry operations on the crystal structure of FadD13 (PDB entry 3r44).^32^ Theoretical scattering profiles and form factors of the FadD13 crystal structure monomer and dimers were calculated and fitted against the experimental data using CRYSOL.^41^ The form factors of the FadD13 monomer and dimer were merged using FFMAKER,^39^ and the fit of the multicomponent mixture and calculations of volume fractions were conducted using OLIGOMER.^40^

### Cross-Linking FadD13 Dimer

The protein sample was diluted to a final concentration of 2.2 mg/mL in cross-linking buffer [50 mM Hepes and 110 mM NaCl (pH 7.5)]. A 20 or 50 mM stock solution of disuccinimidyl suberate (DSS) was prepared in DMSO immediately prior to use. The DSS stock was added to the protein sample to a final concentration of 2 mM DSS (50-fold molar excess). The mixture was incubated at room temperature for 30 min. Thereafter, the reaction was quenched by addition of 1 M Tris-HCl to a final concentration of 50 mM. The sample was centrifuged in a benchtop mini centrifuge to remove debris, and the supernatant was concentrated (VivaSpin 4, 30/50 kDa cutoff). The concentrated sample was subsequently run over a gel filtration column (10/300 Superdex 200 Increase, 0.5 mL/min), and the fraction corresponding to the cross-linked dimer was collected and concentrated (VivaSpin 4, 30 kDa cutoff) to a final concentration of 5–6 mg/mL.

### Liposome Preparation

All lipids were purchased from Avanti Polar Lipids. *E. coli* polar lipid extract (100600C), cardiolipin (18:1, 710335C), and phosphocholine (16:0–18:1, 850457C) were mixed to yield a final 20 mM liposome preparation including 0.5% NBD-PC (18:1–12:0, 810133). When the substrate palmitic acid (Sigma) and/or cardiolipin was included, the amount of *E. coli* polar extract or phosphocholine was adjusted to give a final lipid concentration of 20 mM.

The solvent was evaporated from the lipid mixtures under a stream of N_2_ gas for ∼3 h. The resulting lipid film was hydrated with hydration buffer [50 mM Hepes and 150 mM NaCl (pH 7.3)]. The sample was vortexed extensively and frozen and thawed three times; thereafter, the suspension was pushed through a 1 mL extruder (Avestin) with a 0.1 μm membrane 21 times.

### Sucrose Density Gradient

Sucrose stocks of 3, 2, 1, 0.7, and 0.3 M were prepared in hydration buffer [50 mM Hepes and 150 mM NaCl (pH 7.3)]. The protein:liposome ratio in the sample was 1:10; 10 mM liposomes and 1 mM protein were mixed with 3 M sucrose to yield a final sucrose concentration of 1.6 M. The sucrose density layers were pipetted into a 4 mL Ultra-Clear centrifuge tube (Beckman Coulter) using a Hamilton syringe. The layers were pipetted from the top down, adding the denser layers underneath the previous layer. The final gradient consisted of 250 μL of 2 M sucrose, 500 μL of 1.6 M sucrose (the sample), 250 μL of 1 M sucrose, 2500 μL of 0.7 M sucrose, 250 μL of 0.3 M sucrose, and 250 μL of hydration buffer. The sucrose gradient was centrifuged at 50000 rpm for 17 h at 4 °C (SW60 Ti, Beckman Coulter). After centrifugation, the samples were immediately fractionated into ∼290 μL fractions by a Hamilton syringe, from top to bottom.

A 20 μL sample of each fraction was diluted 10 times in hydration buffer, and the absorbance (280 nm) and emission (533 nm) spectra were recorded (Magellan).

An 11 μL sample was mixed with loading dye and loaded onto a 10% or 4–12% Bis-Tris sodium dodecyl sulfate–polyacrylamide gel electrophoresis (SDS–PAGE) gel (NuPAGE, Invitrogen) run at 180 V in MOPS buffer together with a PageRuler Plus prestained protein ladder (ThermoFisher Scientific). The gels were stained by silver staining. The gels were soaked for 2 h in a fix solution (40% EtOH and 10% acetic acid) and washed for 3 × 20 min with 30% EtOH. Thereafter, the gels were washed with a thiosulfate solution (0.02%) for 1 min and then subsequently washed for 3 × 20 s in dH_2_O before a 1 h incubation in AgNO_3_ (0.2%). The gels were washed again, 3 × 20 s with dH_2_O, and developed for 1–10 min in a developing solution (0.0004% N_2_S_2_O_3_, 3% Na_2_CO_3_, and 0.05% H_2_CO). The development was quenched by exchanging the development solution for a stop solution (5% glycine), and the mixture incubated for 5 min prior to a final wash with dH_2_O. The gels were imaged using a Konica Minolta Bizhub c458 instrument, and the gel band density was analyzed by ImageJ.^42^

### Lipid Strips

Lipid strips were purchased from Echelon. The strips were washed with 4 × 5 mL of wash buffer [10 mM phosphate buffer (pH 7.4), 2.7 mM potassium chloride, 137 mM sodium chloride, 0.05% Tween 20, and 0.5% BSA] and blocked with 5 mL of blocking buffer [10 mM phosphate buffer (pH 7.4), 2.7 mM potassium chloride, 137 mM sodium chloride, 0.05% Tween 20, and 1% BSA] for 1 h at room temperature. The strips were washed, 4 × 5 mL for 5 min, and thereafter incubated with a 6 μM protein solution (FadD13 in wash buffer) for 1 h at room temperature. The strips were subsequently washed, 4 × 5 mL for 5 min, and incubated with the primary antibody (mouse anti-His) diluted 1:2000 in wash buffer for 1 h at room temperature. The strips were washed again, 4 × 5 mL for 5 min, and incubated with a secondary antibody (donkey anti-mouse) and diluted 1:2000 for 1 h at room temperature. Afterward, the strips were washed, 4 × 5 mL for 5 min, and detected with 2 mL of a TMB solution.

### Molecular Graphics

Structural figures were made using PyMOL Molecular Graphics System, version 2.2.2 (Schrödinger, LLC). Electrostatic surfaces were generated using the Adaptive Poisson–Boltzmann Solver (APBS) plugin.^43^

## Results and Discussion

### Membrane Association of FadD13

**Figure 1:**
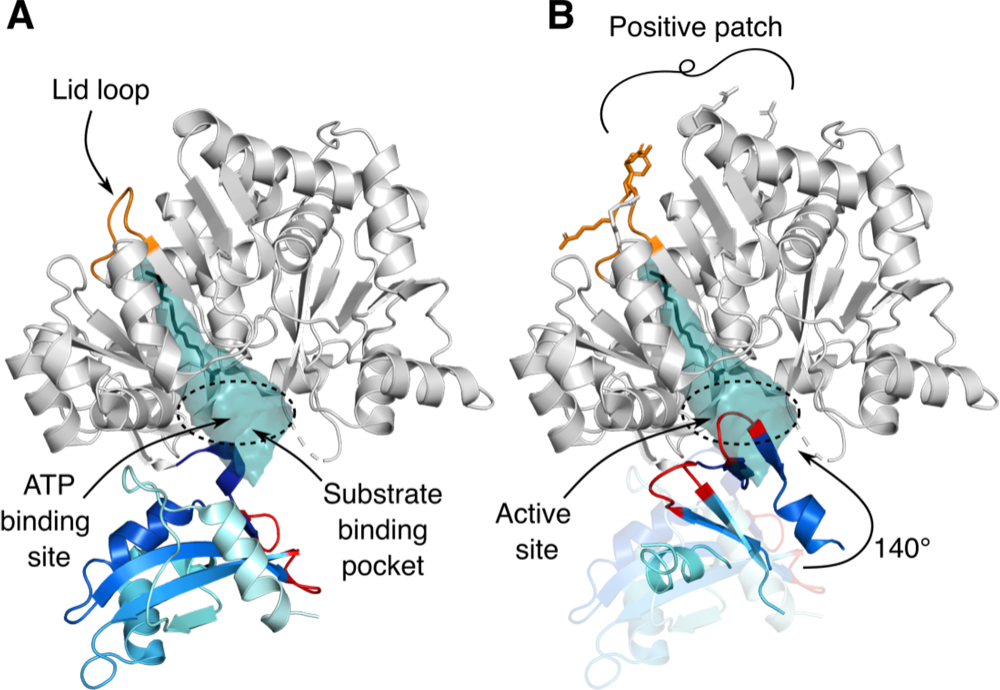
Structural architecture of FadD13. FadD13 (PDB entry 3R44) shown in cartoon representation: N-terminus (gray) and C-terminus (blue–marine–cyan). (A) Substrate binding pocket shown in surface representation (teal). The lid loop (orange) and ATP binding site are indicated by arrows. A myristic acid (C_14_) (black) is overlaid on the structure to highlight the length of the pocket. Loops participating in active site formation are colored red. (B) Residues changed in the mutant variants shown as sticks. The active site is formed by a 140° rotation of the C-terminal domain, illustrated by the C-terminus of ttLC-FACS (PDB entry 1V26)^35^ with a coloring scheme identical to that of the FadD13 C-terminus; the active site-forming loops are colored red. The two C-termini share 41.18% identity and have a very similar fold. Unstructured loops have been removed for the sake of clarity.

FadD13 consists of a large N-terminal domain and a smaller C-terminal domain that is likely to move during catalysis.^32−34^ The active site is formed at the interface between the two domains.^32−34^ There is a large hydrophobic pocket extending from the active site toward a distinct positively charged, arginine rich patch on the surface of the N-terminal domain. The pocket is capped by a flexible lid loop at the protein surface (Figure 1).^32^

We have previously shown that FadD13 is a peripheral membrane protein with soluble as well as membrane-bound populations *in vivo*.^32^ To further understand the dynamic equilibrium between the two enzyme populations, we analyzed the sucrose density co-flotation pattern of FadD13 with liposomes prepared from an *E. coli* lipid extract. The liposomes were supplemented with 0.5% fluorescently labeled phosphocholine (NBD-PC) to detect lipid flotation. The soluble flavoprotein NrdI from *Bacillus cereus*(44) was used as a negative control to verify the assay with and without liposomes.

**Figure 2:**
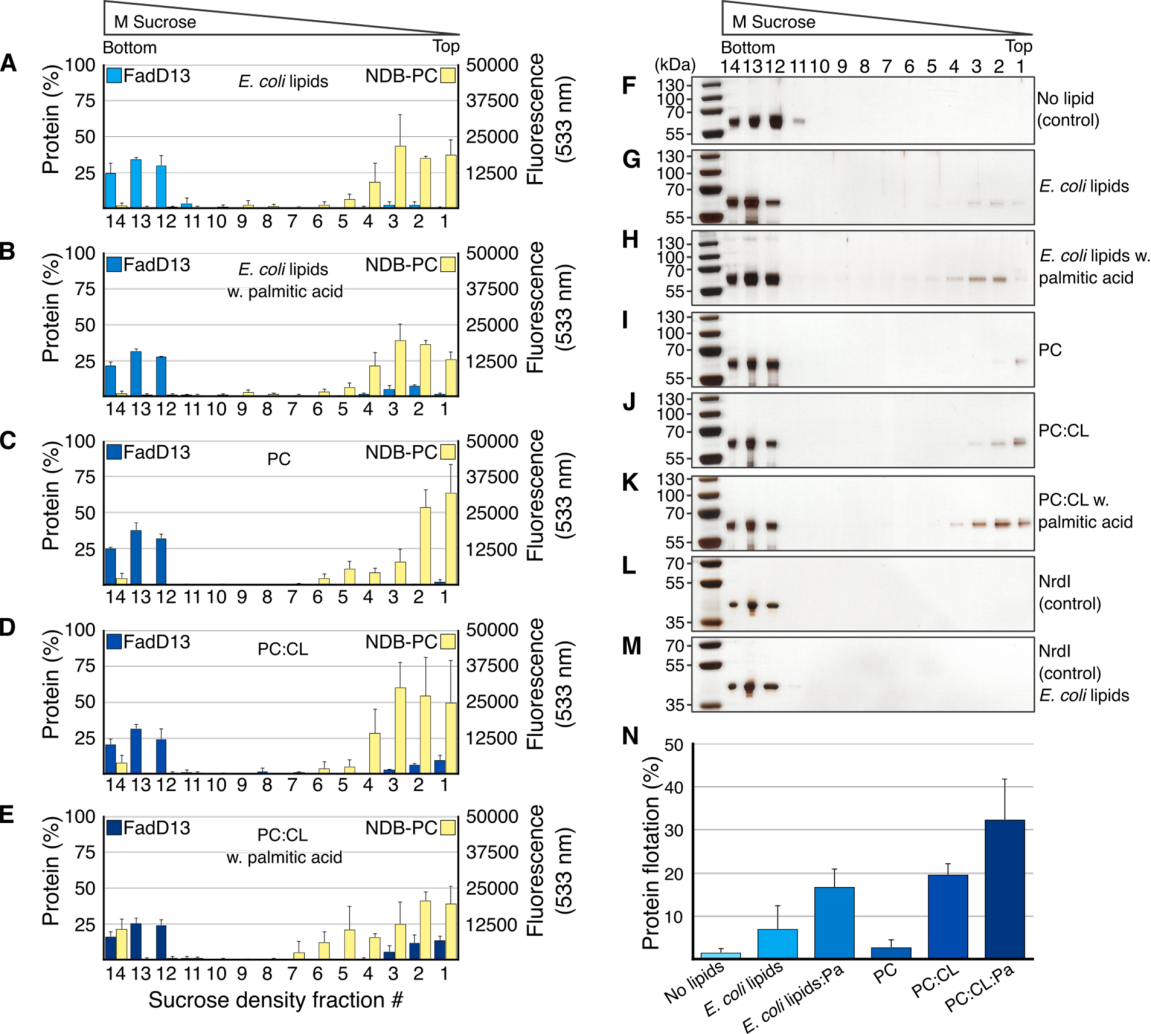
FadD13 liposome interactions. (A–E) SDS–PAGE gel band density vs NBD-PC lipid fluorescence per fraction of the liposome flotation assays. (F–M) Representative silver-stained gels of the liposome flotation experiments. (N) Total amount of co-floating FadD13 (fractions 1–6 combined) vs liposome species: *E. coli* lipid extract with and without palmitic acid (Pa), PC, PC/CL, and PC/CL/Pa liposomes. Averages and standard deviations calculated from three individual experiments, one technical replicate, except the NrdI control, which was made in duplicate, one technical replicate.

Approximately 7% of the total amount of FadD13 was found to co-migrate with the *E. coli* lipid extract liposomes (Figure 2), compared to 2% flotation without liposomes. Because FadD13 presumably exists in an equilibrium between bound and unbound populations, it is expected that the binding of FadD13 to a membrane would be relatively weak. The PPM server^38^ calculates estimates of the energy of transfer for integral and peripheral membrane proteins from water to a lipid bilayer (Δ*G*_transfer_). This binding energy provides a tool for analyzing the spatial arrangement of proteins in relation to a membrane. The energy of binding of FadD13 to a DOPC model membrane is predicted to be −2.5 kcal/mol, which is relatively weak.^38^ The weak binding energy is consistent with the suggested bound–unbound equilibrium. It should be noted that in the sucrose density experiments presented here the populations become separated over time, thus disrupting the equilibrium. Therefore, the amount of protein bound to the liposomes in these experiments is expected to slightly underestimate the true affinity of FadD13 for the liposomes.

Most fatty acyl synthetases have a defined hydrophobic pocket matching the preferred substrate size.^33,35,45,46^ The hydrophobic pocket of FadD13 can house a fatty acid with an acyl chain of approximately 14 carbons (C_14_)^32^ (Figure 1). Intriguingly, the preferred substrate of FadD13 has considerably longer acyl chains; the activity of FadD13 increases with substrate length up to the limit tested (C_26_).^27^ We have previously hypothesized that FadD13 adheres to the membrane via the positively charged surface patch, allowing the membrane to partially house the very-long-chain fatty acid substrates.^32^ If so, addition of a substrate would be expected to anchor the enzyme more tightly to the membrane. Indeed, when 10% palmitic acid (C_16_) was incorporated into the *E. coli* lipid extract liposomes, the level of binding of FadD13 was increased >2-fold to 17% (Figure 2N). Addition of tetracosanoic acid (C_24_) unfortunately proved to be incompatible with liposome preparation.

The inner membrane of *M. tuberculosis* is mainly comprised of the lipid species phosphatidylethanolamine (PE), phosphatidylglycerol (PG), cardiolipin (CL), phosphatidylinositol (PI), and phosphatidyl-*myo*-inositol mannosides (PIM).^47−49^ FadD13 shows a clear preference for negatively charged headgroups such as CL, phosphatidic acid (PA), and phosphatidylinositol 4-phosphate (PI4P) (Figure S1). However, FadD13 does not appear to have significant affinity for the lipid headgroups of PE, PG, PI, or PC.

Phosphatidylinositol phosphates [PI(*x*)P_*x*_] are not known to be constituents of the mycobacterial inner membrane, and PA is not produced in appreciable amounts;^47,48^ it may exist mainly as a precursor of more complex lipids. CL, on the contrary, is one of the major lipids in the mycobacterial inner membrane, constituting approximately 1.2% of the total lipid mass^48^ and known to be involved in many protein–membrane interactions.^50,51^

The polar *E. coli* lipid extract (Avanti) consists of 67% PE, 23% PG, and 10% CL. Because FadD13 does not show significant binding to either PE or PG lipid headgroups, it is likely that the binding of FadD13 to the liposomes is mainly achieved by its affinity for CL.

To further assess the impact of CL and a substrate on FadD13 membrane association, we compared the affinity of FadD13 for pure PC liposomes, PC liposomes supplemented with 30% CL (PC/CL), and PC/CL liposomes supplemented with 10% palmitic acid (PC/CL/Pa), As expected, FadD13 showed no significant affinity for the zwitterionic PC liposomes (3%). However, incorporation of 30% CL to the liposomes increased the level of co-flotation almost 7-fold to 20% (Figure 2N). This is 3 times the level of binding of FadD13 to the *E. coli* lipid extract liposomes that contained 10% CL. Incorporation of palmitic acid into the PC/CL liposomes further increased the level of co-flotation of FadD13 to 32% (Figure 2N). The PC/CL/Pa liposomes detected in the bottom fraction of the co-flotation experiments (Figure 2E) are likely an artifact of fractionation due to the propensity of PC/CL liposomes to float above the top layer, making fractionation difficult.

These data suggest that FadD13 associates primarily with negative lipids such as CL and that the enzyme is further anchored to the membrane by acquisition of a substrate.

### Multimeric Arrangement of FadD13

The multimeric arrangement of FadD13 has been ambiguous. FadD13 is a 55 kDa protein but elutes around 80–90 kDa on a gel filtration column, between the expected size of a monomer and that of a dimer. The gel filtration peak typically has a small shoulder, sometimes a split peak, indicating the existence of more than one oligomeric state (Figure S2).

An analysis of the quaternary structure of FadD13 (PDB entry 3R44)^32^ by the PDBe PISA server^52^ places FadD13 in a gray area for multimerization with potential dimeric arrangements that may or may not be stable in solution. There are reports of both monomeric and dimeric FACS in the literature,^35−37^ and from the structure of FadD13, it is possible to generate two dimers based on crystal-related symmetry: one C-terminal dimer, where the dimer interface lies near the active site at the C-terminal hinge region, and one N-terminal dimer, where the dimerization interface lies near the large positively charged surface patch.^32^

**Figure 3:**
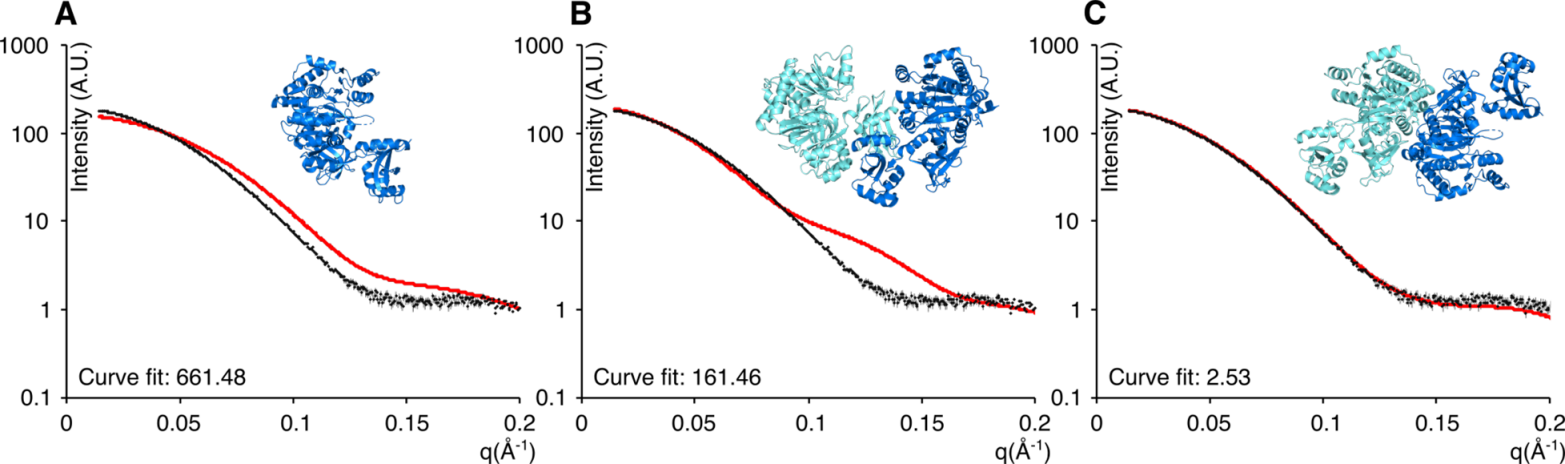
Comparison of the theoretical curves with experimental SAXS data. Experimental data (black) vs (A) theoretical monomer curve (red), (B) theoretical C-terminal dimer curve (red), and (C) theoretical N-terminal dimer curve (red).

To resolve the ambiguity regarding the oligomeric state of FadD13, we performed SAXS of FadD13 in solution. Comparison between the experimentally obtained curves and theoretically calculated curves of the monomer, C-terminal dimer, and N-terminal dimer unambiguously shows that FadD13 adopts the N-terminal dimeric arrangement in solution (Figure 3).

The proposed N-terminal dimer is structurally similar to previously determined crystal structures of the *Thermus thermophilus* FACS (ttLC-FACS) (PDB entry 1ULT, UniProt entry Q5SKN9).^35^ The interaction interfaces of the two dimers are quite similar; however, the FadD13 dimer interface does not share the domain swap present in ttLC-FACS, and therefore, the interface is smaller and slightly shifted compared to the ttLC-FACS interface (Figure S3). The FadD13 dimer is more extended compared to the apo structure of ttLC-FACS, which gives a distinct difference in quaternary shape. This difference is apparent in the SAXS data as the theoretically calculated curves of FadD13 fit significantly better to the experimental data compared to the theoretical curves calculated from ttLC-FACS (Table 1).

**Table 1:** SAXS Data

	wild-type FadD13	hydrophobic variant	negative variant
Invariant Parameters			
*D*_max_ (Å)	116	113	114
*R*_g_ (Å)	33.67	31.98	32.13
Porod volume (Å^3^)	177016	89458	88936
Curve Fits (χ^2^)			
*T. thermophilus* (1ULT)	5.32	–	–
C-terminal dimer	161.46	170.86	81.05
N-terminal dimer	2.53	141.52	61.75
monomer	661.48	52.41	43.21
“open conformation monomer”	689.62	46.12	32.87
dimer/monomer	–	21.84	15.32
dimer/“open conformation monomer”	–	3.83	2.54

**Figure 4:**
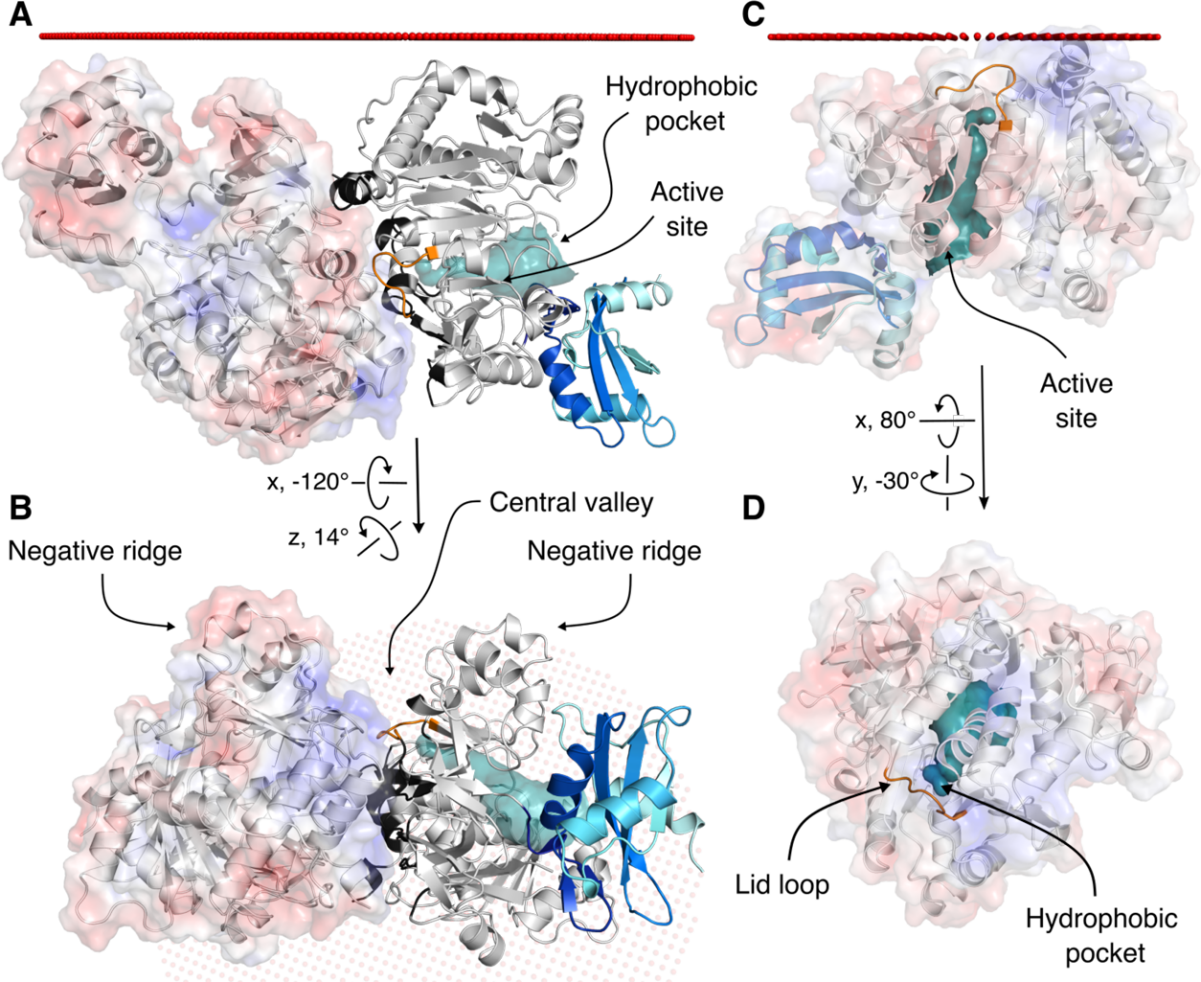
Dimeric and monomeric assemblies of FadD13. (A) N-Terminal dimer of FadD13 in relation to a modeled membrane, illustrated by spheres (red). Left monomer shown with a transparent electrostatic surface representation calculated by the APBS tool in PyMOL (red, −5 kT/e; blue, 5 kT/e). Right monomer shown by a cartoon representation: N-terminal domain (gray), C-terminal domain (blue–marine–cyan), and hydrophobic pocket (teal). Residues participating in the dimer interaction (black) and the lid loop (orange) covering the entrance to the hydrophobic pocket. (B) The dimer is rotated 120° from the viewer, viewed from the cytoplasm, showing the positively charged central valley formed between the monomers flanked by two large negative ridges. (C) Monomer of FadD13 in relation to a modeled membrane (red spheres). FadD13 is calculated to interact with the membrane via the positively charged patch on the N-terminal surface, which places the lid loop and entrance to hydrophobic pocket in a favorable position for acquisition of a substrate from the membrane. (D) The monomer is rotated 80° toward the viewer, viewed from the proposed membrane position.

The FadD13 dimer interaction surface, calculated to 1757 Å^2^ by the PISA server, is less hydrophobic than expected for a permanent dimeric arrangement with a Δ^i^*G* calculated to be −0.8 kcal/mol. The interaction is dominated by salt bridges and hydrogen bonding and involves 44 residues in total: Met^1^-Lys^2^, Trp^6^-Met^7^, Arg^9^-Gln-Arg^11^, Thr^13^-Val-Ser-Pro-Arg^17^, Glu^179^, Ser^183^, Ser^186^, Ala^189^-Ser-Thr-Ile-Asp-Val-Arg-Tyr-Arg^197^, Ala^219^-Met-Arg^221^, Leu^314^, Glu^316^, Arg^327^-Ala-Thr-Met-Phe-Thr-Asp^333^, Glu^345^, Lys^354^-Ser-Asp^356^, Gly^376^-Trp^377^, and Glu^389^-Gly^390^ (Figure 4). Approximately half of these 44 residues are also involved in the positively charged surface patch proposed to be involved in membrane binding.^32^ In particular, Lys^2^, Arg^9^, Arg^11^, Arg^17^, Arg^195^, Arg^197^, and Arg^221^ together with neighboring residues Arg^29^, Arg^199^, Arg^244^, and Arg^272^ contribute to the distinct positive charge of this area (Figures 1 and 4). The last four arginines are not involved in the dimer interaction; instead, they participate in a valley formed between the two monomers (Figure 4). This valley, termed the central valley by Hisanaga et al.,^35^ has been proposed to be a membrane anchoring valley. The valley does have a distinct positive charge, but it is flanked by two large negatively charged ridges. It would be energetically unfavorable for these ridges to protrude into the membrane surface. Thus, the central valley appears to be inaccessible to the membrane. Additionally, the dimeric arrangement blocks the entrance to the hydrophobic substrate-housing pocket, likely incompatible with the observed substrate specificity.

Indeed, modeling the spatial arrangement of the FadD13 dimer in the proximity of a model membrane does not provide a plausible mode of binding to the membrane. However, when the monomer is modeled in the proximity of a membrane, the PPM server suggests it would successfully interact with the membrane via the positively charged surface patch. These results suggest that membrane binding via the central valley is unlikely (Figure 4).

### Cross-Linking FadD13 Dimers

**Figure 5:**
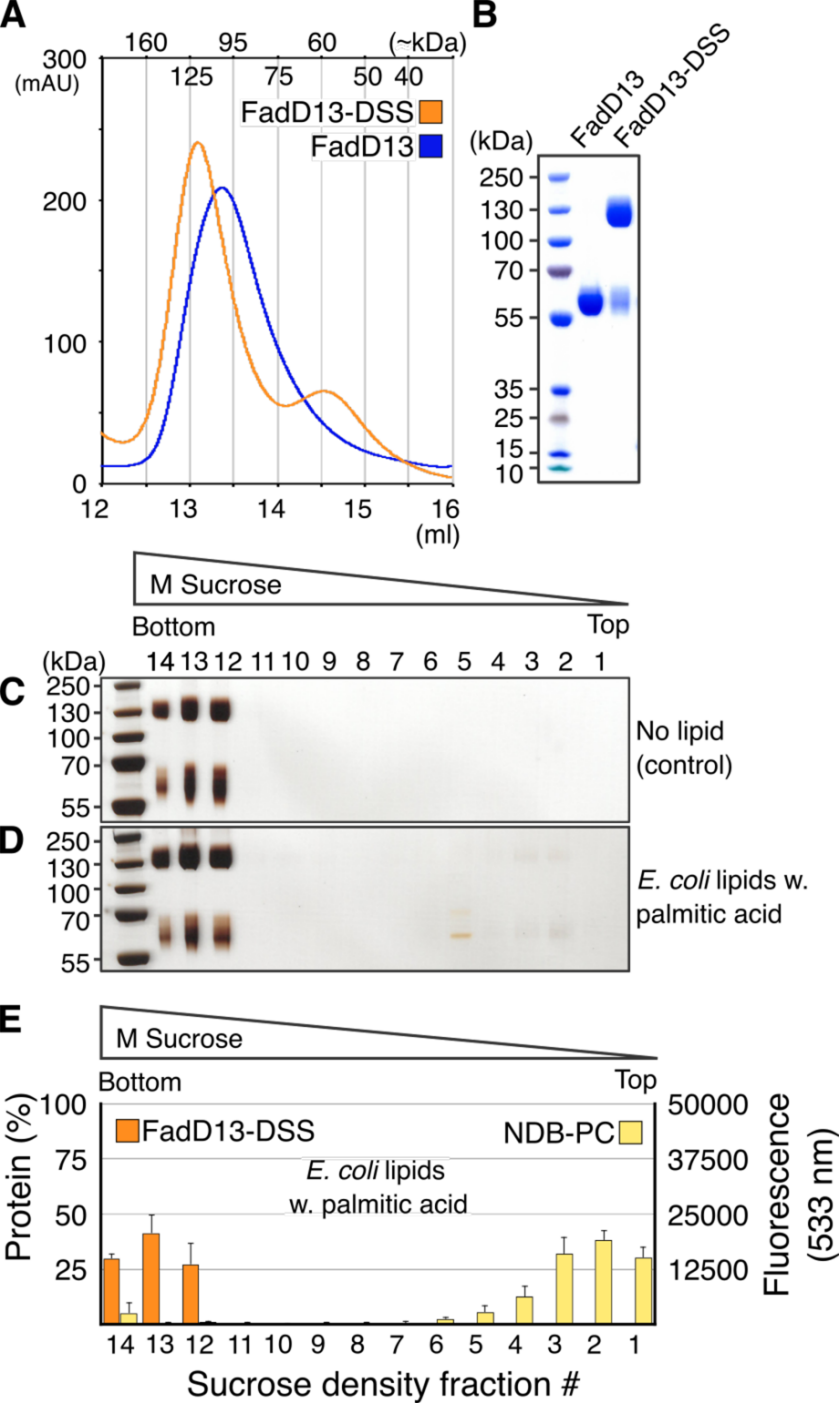
Cross-linked FadD13 dimer liposome interactions. (A) Gel filtration chromatogram of FadD13 and cross-linked FadD13 dimer (FadD13-DSS). (B) Coomassie-stained SDS–PAGE gel of FadD13 and FadD13-DSS. (C and D) Representative silver-stained gels of liposome flotation experiments. (E) SDS–PAGE gel band density vs NBD-PC lipid fluorescence per fraction of the liposome flotation assays. Standard deviations calculated from four individual experiments, one technical replicate.

To understand how dimerization of FadD13 influences membrane association, we cross-linked the FadD13 dimer with disuccinimidyl suberate (DSS). Cross-linking the FadD13 dimer successfully resolved the sample into dimeric and monomeric populations that could be separated by gel filtration. The peaks correlate well with the expected sizes of a dimer and monomer (Figure 5).

The cross-linked FadD13 dimer did not appear to co-migrate with *E. coli* lipid extract liposomes supplemented with 10% palmitic acid. Only a negligible amount of the FadD13 dimer could be detected in the liposome-containing fractions (Figure 5). However, a portion of non-cross-linked FadD13 remained in the sample after cross-linking and subsequent purification (Figure 5). The portion of non-cross-linked material was estimated to ∼24% by gel band density analysis. Consistent with previous results, the non-cross-linked protein appeared to be capable of migrating with the liposomes as some non-cross-linked enzyme was found in the top liposome-containing fractions (fractions 1–6). Due to the small amount of non-cross-linked protein, quantification was not attempted (Figure 5).

DSS acts on primary amines to form stable, noncleavable amide bonds. It is possible that such amide bonds would have a negative effect on the membrane association of the monomers and thus result in less of the non-cross-linked protein fraction bound to the membrane than expected. However, it is less likely that the amid bonds *per se* would have a similar effect on the membrane association of the dimer because our modeling data indicate that if there were a dimer–membrane interaction, it would have to be governed by other mechanisms, and not predominantly by charge. It is likely that such interaction would require a large structural rearrangement of the dimer to allow access to the hydrophobic pocket, etc.

Thus, these observations taken together imply that the cross-linked FadD13 dimer, as observed in the crystal structure and our SAXS analysis, is unable to bind to and co-migrate with the liposomes due to the inability of the dimers to dissociate or rearrange (Figure 5).

### Mutant Variants of FadD13

To further assess the interactions involved in both dimerization and membrane association, we utilized a set of mutant variants with changes to central residues of the positively charged surface patch, proposed to be involved in membrane association. A hydrophobic variant (R9A/R17A/R195A/R197A/R244A) and a negative variant (R9E/R17D/R195E/Y196A/R197D/R244D) was used.

The variants showed slightly lower expression than wtFadD13, and both variants were prone to aggregation, resulting in a poor yield from the gel filtration purification step(s), especially for the negative variant. However, both were stable upon isolation, and it was possible to purify them using the same protocol as developed for wtFadD13.

The mutant variants eluted with one major peak corresponding to a size of approximately 50 kDa, suggestive of a monomeric arrangement. The negative variant had a distinct shoulder on the left-hand side of the major peak, likely corresponding to a fraction of dimeric protein (Figure S4).

### SAXS Analysis of FadD13 Variants

To characterize the effect of these mutations on tertiary and quaternary structure, the solution scattering was measured. While the distance parameters radius of gyration and maximum intraparticle distance (*D*_max_) remained consistent with those expected from a dimeric FadD13, the calculated particle volumes of the variants were roughly half of that of the wild type, supporting a mainly monomeric state in the variant samples (Table 1).

**Figure 6:**
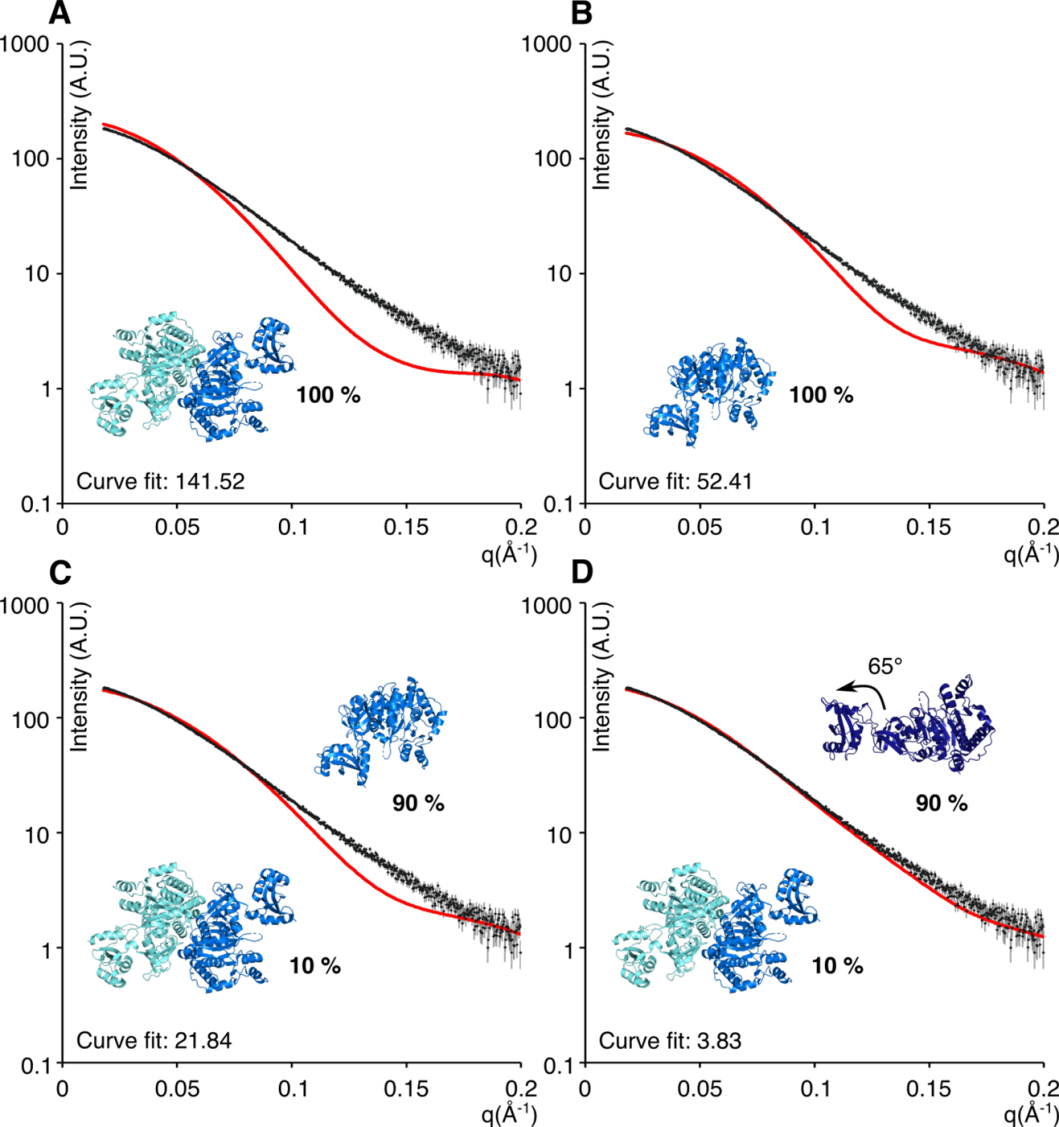
Comparison of experimental SAXS data of the hydrophobic variant (black) with theoretical curves (red) of (A) the FadD13 dimer, (B) the FadD13 monomer, (C) the FadD13 dimer/monomer mixture, and (D) the FadD13 dimer/“open conformation monomer” mixture. A comparison of all curve fits can be found in Table 1.

As the invariant parameters seemed to indicate the presence of FadD13 in both monomeric and dimeric states, and because the scattering data did not appear to equate to the monomeric or dimeric state of the protein (Figure 6A,B), the program OLIGOMER was utilized to investigate a possible equilibrium between the two. The calculated volume fractions revealed that the equilibrium was dominated by a 90% volume fraction of monomeric protein. The calculated monomer–dimer equilibrium does, however, not fully explain the scattering data (Figure 6C). Therefore, we speculate that the “free” monomers are in a conformation slightly different from those bound in a dimer. To allow the creation of an average solution conformation, an extended “open monomer model” of the hydrophobic variant was created by allowing the C-terminal domain to pivot around the hinge region (as observed and described in ref (34)) in steps of 5°. Each structure was fitted in OLIGOMER alongside the N-terminal dimer. The volume fractions remained constant, while a clear improvement in χ was observed with the best fit observed for a rotation of 65° around Lys^392^ (Table 1 and Figure 6D).

### Membrane Association of FadD13 Variants

**Figure 7:**
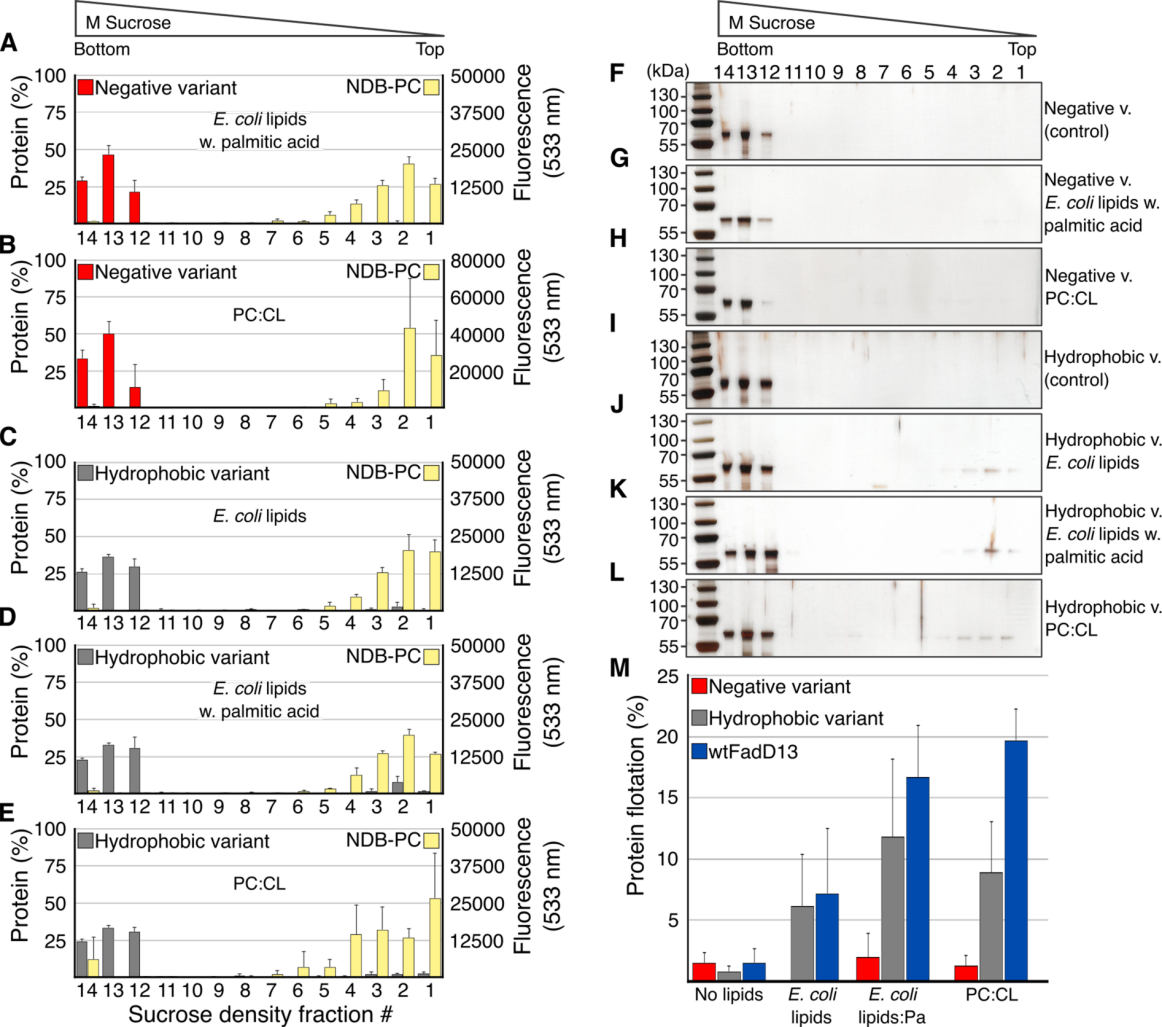
Liposome interactions of the FadD13 variants. (A–E) SDS–PAGE gel band density vs NBD-PC lipid fluorescence per fraction of the liposome flotation assays. (F–L) Representative silver-stained SDS–PAGE gels from the liposome flotation assays. (M) Total amount of protein in top fractions (fractions 1–6 combined) co-floating without lipids, with *E. coli* lipid extract liposomes, with and without palmitic acid, and with PC/CL liposomes. Averages and standard deviations calculated from three individual experiments, one technical replicate, except the negative variant with PC/CL, which was made in duplicate, one technical replicate.

The hydrophobic variant appeared to maintain its ability to bind both liposomes and substrate. Approximately 6% of the hydrophobic variant was found to co-migrate with the *E. coli* lipid extract liposomes, and when palmitic acid was added, the level of binding was increased 2-fold to 12% (Figure 7), on par with the increase in the level of binding observed for wtFadD13 in the presence of substrate (see above).

**Figure 8:**
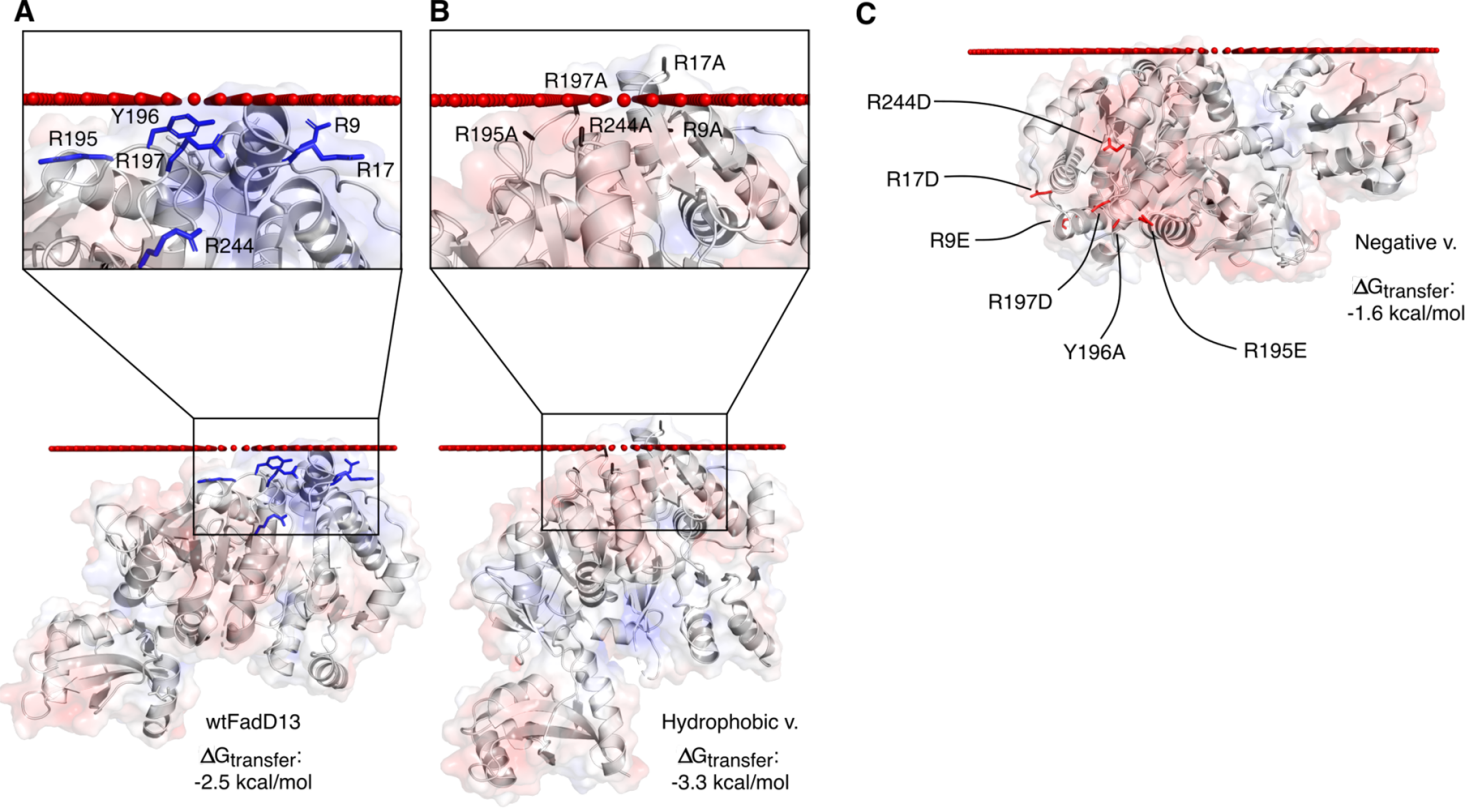
FadD13 variants modeled in the proximity of a lipid bilayer. (A) Modeled membrane interaction of wtFadD13, with important residues (blue sticks) highlighted in the inset. (B) Modeled membrane interaction of the hydrophobic variant, with mutated residues (gray sticks) highlighted in the inset. (C) Modeled membrane interaction of the negative variant, with mutated residues shown as red sticks. Transparent electrostatic surface representation calculated by the APBS tool in PyMOL (red, −5 kT/e; blue, 5 kT/e), positioned in the proximity of the bilayer calculated by the PPM server.

The PPM server suggests that the hydrophobic variant should have a slightly stronger binding to a lipid bilayer compared to that of wtFadD13 (−3.3 kcal/mol compared to −2.5 kcal/mol) (Figure 8). These numbers are indicative of weak membrane interactions and are calculated for a DOPC model membrane. Thus, they do not take into account the existence of potential CL-enriched areas, which may explain the slightly stronger interaction observed for wtFadD13 *in vitro*. The levels of co-flotation of wtFadD13 and the hydrophobic variant with *E. coli* lipid extract (10% CL) are very similar (7 ± 5% compared to 6 ± 4%). However, when the amount of CL was increased to 30%, wtFadD13 had a larger population bound to the liposomes compared to the hydrophobic variant (20 ± 3% compared to 9 ± 4%) (Figure 7). We have previously shown that it is possible to wash FadD13 of the membranes by an increase in ionic strength, a high salt concentration, or an alkaline pH.^32^ Taken together with the results presented here, this indicates that charge is indeed an important factor for membrane association of the wild-type protein.

In addition, the PPM server predicts the negative variant will have a weaker membrane interaction compared to that of wtFadD13 (−1.6 kcal/mol compared to −2.5 kcal/mol). The negative variant is not expected to retain the ability to adhere to the membrane via the N-terminal surface patch (Figure 8). These predictions are confirmed by the sucrose flotation assays; the negative variant did not appear to co-migrate with the liposomes regardless of whether the substrate was present or if the amount of CL was increased (Figure 7).

## Conclusions

*M. tuberculosis* continues to be a major threat to world health, and a more detailed understanding of the enzymes involved in its lipid metabolism is important in our battle against this significant human pathogen. FadD13 is one of 34 mycobacterial FACS and has been suggested to be involved in remodeling of the mycobacterial outer membrane in response to acidic conditions.^29−31^

Structural analysis and modeling of the FadD13 monomer support the hypothesis that FadD13 adheres to the membrane via a distinct, positively charged patch on its N-terminal surface. Here we show that FadD13 has an affinity for liposomes and that the affinity increases with the amount of negatively charged CL present, consistent with the proposed mode of membrane attachment. We also conclude that FadD13 is a dimer in solution. The dimer interface partially covers the suggested membrane binding patch on the N-terminal surface, and the dimer itself does not provide a plausible alternative mode of membrane binding. Cross-linking the dimer efficiently abolished membrane adhesion, presumably due to its inability to dissociate. We speculate that the dimeric arrangement in solution exists to prevent unwanted interactions with other cellular components and that it is not the active form of the enzyme.

The structure of FadD13 reveals a large hydrophobic pocket extending from the active site to the N-terminal patch, proposed to be involved in membrane binding. The pocket has the capacity to harbor an acyl chain of approximately 14 carbons (C_14_) but would not be able to house the very-long-chain (>C_26_) substrates preferred by the enzyme. Other *M. tuberculosis* FACS enzymes face similar conundrums. FadD32, for example, adenylates meromycolic acids (C_48_–C_64_), but its hydrophobic pocket cannot accommodate these very long acyl chains either; it has been suggested that it utilizes its partner protein PKS13 to house the protruding acyl chains.^37^

We have previously proposed that FadD13 may utilize the membrane interaction to house parts of the acyl chains that do not fit within the enzyme.^32^ Consistent with this notion, we show that incorporation of a substrate in the liposomes increases the amount of protein observed to co-migrate with them. Thus, it seems that addition of a substrate further anchors the enzyme to the liposome surface.

Changing the physical properties of the positive patch and dimer interface region changes dimerization behavior as well as lipid affinity. Both FadD13 variants were predominantly monomeric but present striking differences in membrane affinity. The negative variant lost its ability to bind liposomes, presumably due to the repulsion by the negative charges; moreover, addition of a substrate did not induce co-migration with the liposomes of this variant. The hydrophobic variant retained the ability to bind both liposomes and substrate. When the amount of CL in the liposomes was increased, the membrane-bound population of the hydrophobic variant did not increase. Liposome binding by wtFadD13 on the contrary was increased several-fold when the CL content was increased. We interpret this observation as charge being an important factor for membrane association of the wild-type protein and speculate that FadD13 might adhere to CL rich areas within the membrane

**Figure 9:**
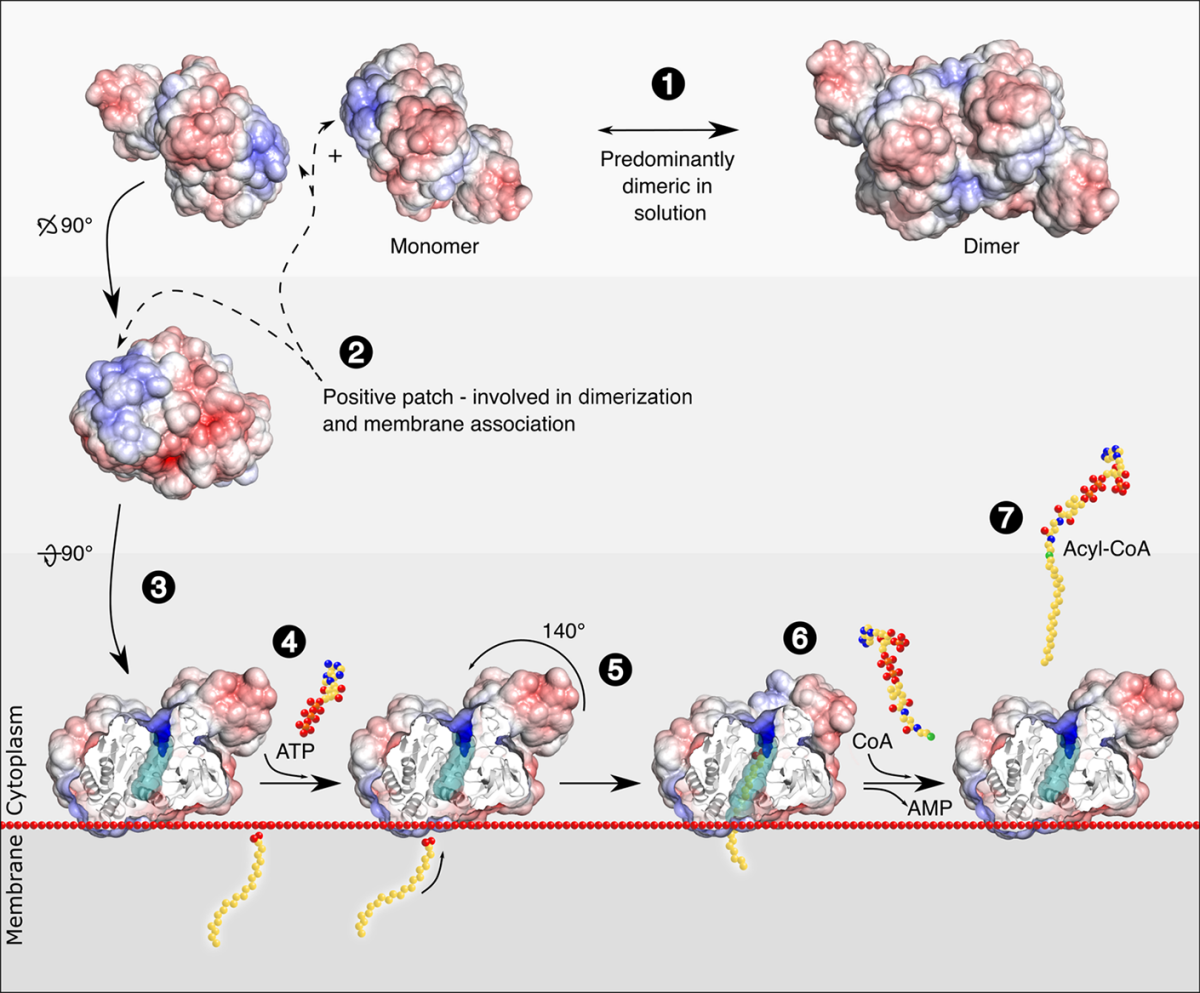
Proposed mechanism of fatty acid activation by FadD13. FadD13 is predominantly dimeric in solution (1), but monomeric at the membrane, and it utilizes a positively charged surface patch to adhere to the net-negative membrane (2 and 3). Binding of ATP (4) induces a conformational change, and the C-terminal domain is rotated 140°, creating the active site together with residues of the N-terminal domain (5). The very-long-chain fatty acid substrate is likely to originate from the membrane and may partially reside within the membrane during catalysis (6). After attachment of CoA, the activated fatty acyl-CoA may be released (7). FadD13 (PDB entry 3R44) is shown with an electrostatic surface representation, calculated by the APBS tool in PyMOL (red, −5 kT/e; blue, 5 kT/e). The hydrophobic pocket is colored teal.

In light of these observations, we propose that FadD13 exists in a dimer–monomer equilibrium where the dimeric state dominates in solution while the monomeric state binds to the membrane, utilizing the positively charged patch as an anchor. This would allow FadD13 access to fatty acyl substrates, likely to reside within the membrane, and allow the membrane to house the protruding acyl chains of the very-long-chain substrates of the enzyme (Figure 9).

## Supporting Information Available

Lipid strips assay, gel filtration chromatogram of wtFadD13, comparison of the dimer interface within FadD13 and ttLC-FACS, and a comparison of the gel filtration chromatograms of wtFadD13 and the variants (PDF)

## Accession Codes

FadD13, P9WQ37; ttLC-FACS, Q5SKN9.

## Abbreviations

TB: tuberculosis
WHO: World Health Organization
CoA: coenzyme A
FACS: fatty acyl-CoA synthetases
AAE: acyl activating enzymes
SAXS: small-angle X-ray scattering
PPM: Positioning of Proteins in Membrane server
DOPC: dioleoylphosphatidylcholine
wtFadD13: wild-type FadD13
NBD-PC: fluorescently labeled phosphocholine
PE: phosphatidylethanolamine
PG: phosphatidylglycerol
CL: cardiolipin
PI: phosphatidylinositol
PIM: phosphatidyl-*myo*-inositol mannosides
PA: phosphatidic acid
PI4P: phosphatidylinositol 4-phosphate
PI(*x*)P_*x*_: phosphatidylinositol phosphates
PA: palmitic acid
ttLC-FACS: *T. thermophilus* FACS
DSS: disuccinimidyl suberate.
