# Supplementary material for: Solution and Membrane Interaction Dynamics of Mycobacterium tuberculosis Fatty Acyl-CoA Synthetase FadD13
Source: Biochemistry. 2021 Apr 29;60(19):1520–32. doi: 10.1021/acs.biochem.0c00987 (PMC8253482; doi:10.1021/acs.biochem.0c00987)
Supplement: Supplementary file 1 — bi0c00987_si_001.pdf [file bi0c00987_si_001.pdf]

# Solution and Membrane Interaction Dynamics of *Mycobacterium tuberculosis* Fatty Acyl-CoA Synthetase FadD13

Camilla A. K. Lundgren[1, 2], Michael Lerche[1, 3], Charlotta Norling[1, 4] and Martin Högbom[1*]

[1]Department of Biochemistry and Biophysics, Stockholm University, Stockholm, Sweden.

AUTHOR INFORMATION

**Corresponding Author**

*Correspondence should be addressed to Martin Högbom, e-mail: hogbom@dbb.su.se.

**Present Addresses**

[2]Sir William Dunn School of Pathology, University of Oxford, Oxford, UK. [3]AstraZeneca AB, Mölndal, Sweden. [4]Roche Diagnostics Scandinavia AB, Solna, Sweden.

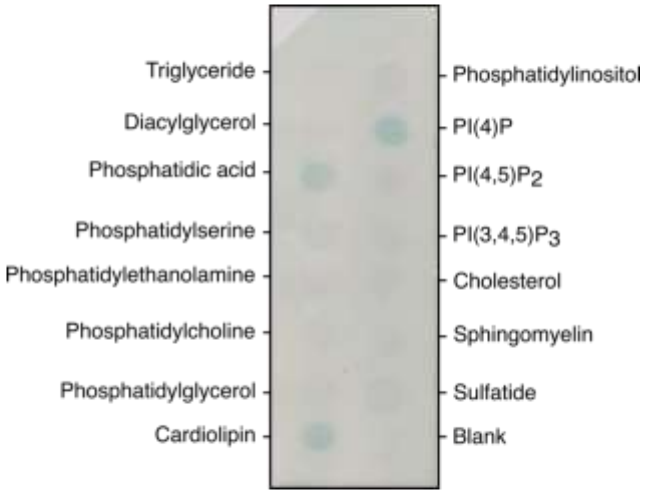


**Supplemental figure 1**. Lipid strip assay. Representative image of three individual experiments.

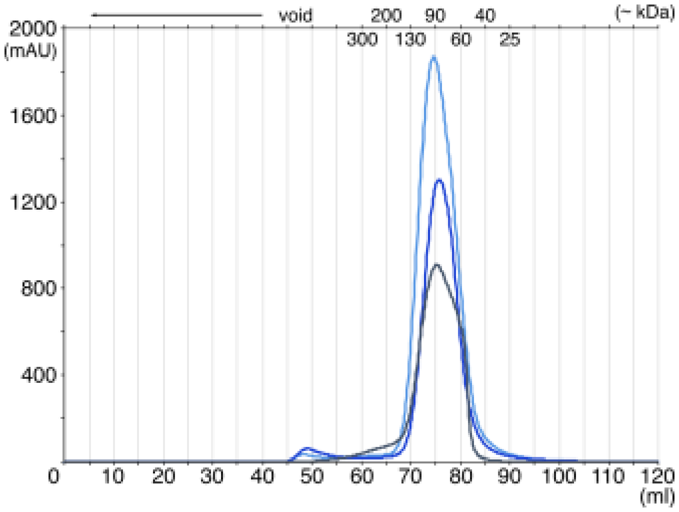


**Supplemental figure 2.** Gel filtration chromatogram of FadD13. Three individual experiments shown, representative of n ≥ 10 experiments.

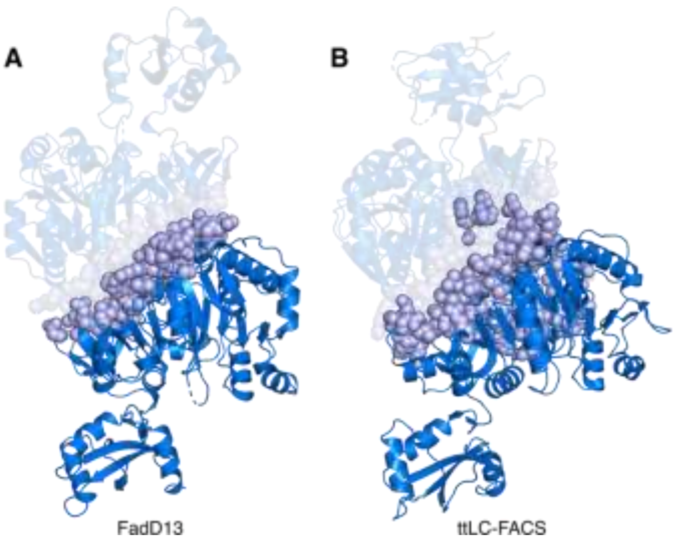


**Supplemental figure 3.** A comparison between the interface of A) FadD13 dimer (PDB entry 3R44) and B) ttLC-FACS dimer (PDB entry 1ULT). Interface residues highlighted as spheres; one monomer shown as transparent cartoon for clarity.

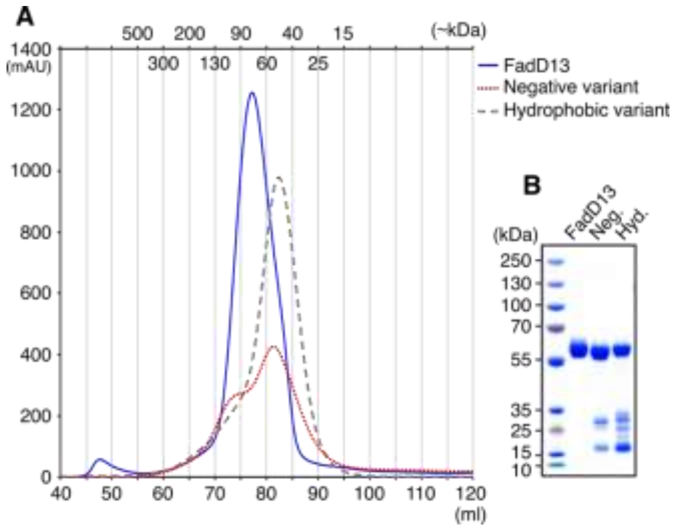


**Supplemental figure 4.** Purification of FadD13 variants vs. wtFadD13. A) Gel filtration chromatogram of FadD13 (blue, full), negative variant (red, dotted) and hydrophobic variant (gray, broken). B) Coomassie stained SDS-PAGE gel of wtFadD13 and FadD13 variants.
